# On the Management of the Sick in the Prisons of Norway

**Published:** 1844-01

**Authors:** 


### ART. III.

*Om Sygepleien i Straffeanstalterne i Norge.* Ved Prof. F. HOLST.—*Christiania*, 1841. 8vo, pp. 112.

*On the Management of the Sick in the Prisons of Norway.* By Prof. FRED. HOLST.—*Christiania*, 1841.

IT was not till the recent changes in prison discipline, which have been adopted in Europe and America, that public attention was directed to the sanatory state of prisoners in various houses of correction. In this respect however the Norwegian goals have remained unnoticed, save by a short essay from the hands of the author of the present pamphlet, in 1828. In the month of September, 1837, a commission for this purpose, was formed, and Professor Holst was included among its members. Its aim was to investigate the present state of the houses of detention in Norway, and to propose such measures of reform, as would be most likely to ameliorate their condition.

There exist in Norway two kinds of prisons. The more severely disciplined are appropriated to the greater offenders, of the male sex only, who are denominated slaves, and their place of confinement slave-prisons, (*slaverier;*) the other kind are *tugthuse* or penitentiaries, and contain the lesser criminals of the male sex, with all female convicts. The slave-prisons may be considered as more immediately under the military; the penitentiaries under the civil power. In the year 1840, the total number of criminals of both sexes was, singularly enough, 1840, viz. of men 1528, of women 312, and of this number 1203 males were in the slaverier, or in other words undergoing the severer degrees of penalty. The entire population of Norway is 1,276,300, which gives a proportion of one criminal in 694 inhabitants.

Previous to speaking of the treatment of the sick, Dr. Holst mentions several circumstances regarding the Norwegian prisons, which must naturally influence the health of their inmates. They are often, he says, situated in the immediate neighbourhood of the ditches of the fortress which are usually filled with stagnant water, and the cells themselves are low, and often much too confined to admit of a free circulation of air. In the prisons of Bergen, in particular, we observe by Dr. Holst's tables, that 133 and 152 cubic feet of air are all that is appropriated to each individual in his sleeping cell. Besides this there is a great want in the

Norwegian prisons of proper space within the walls for the exercise of the prisoners in the open air, though in that country this is an object of much less importance than in England, as the greater part of the slaves are employed in chains, upon the public works, without the walls of the goal. The slaves (slaver) rise in summer at 4 A.M., and in winter at 7 A.M. and retire to rest during the former season at 9, and in winter at 8 P.M. For those confined in the penitentiaries, the working period averages about eleven hours per day. The slaves receive in all the prisons 2lb. of bread daily, as also two skillings in money, with which they are allowed to purchase eatables and liquor, (chiefly corn-brandy,) from the shops which exist in all prisons, excepting in that of Vardoehuus. At this last-named spot, the prisoners are allowed the daily sum of five skillings, without any bread, and for this money they purchase food from the stores of the fortress at a moderate rate. Their meat, however, is in a raw state, and the cooking of it within their cells occasions much accumulation of matter prejudicial to health, though their food itself is in general excellent in quality. Spirits are allowed to be sold openly in all prisons, excepting in those of Aggershuus and Trondhjem. Dr. Holst complains that the prison dress is not sufficiently warm for those that work out of doors during the winter, nor is much care taken about its being regularly cleaned and changed. Two prisoners in general occupy one bed, but in the slave-house (slaverier) of Fredrikstad, one bed suffices for three, and in some prisons, the beds are placed like berths in ships of passage, one tier above the other. It must be extremely difficult if not impossible, to preserve cleanliness in a room, where the prisoners not only dwell and work during the day, but also prepare their food and consume it. The arrangements for the cleansing the persons and linen of the inmates are also but little attended to, though in most prisons they are allowed the privilege of sea-bathing. In the district of Vardoehuus, however, few will avail themselves of this privilege, as the water is too cold for the most daring, even in the height of summer. The *slaves* are chiefly employed upon public works, such as quarrying, cleansing the ditches of the fortress, or the streets of the town, and in Vardoehuus are even engaged in the sea fishery, while, at other times, they work within the walls of their prisons at various trades. But as at present constructed, the slave-prisons are by no means adequate to provide labour for all their inmates, for out of 100 able-bodied men, it appears that not less than 26 pass their time in idleness. In the penitentiaries, the men are kept to lighter kinds of labour, as are also the female prisoners, and many of the latter officiate as cooks.

From these preliminary observations Dr. Holst passes to the immediate object of his essay. By a well-arranged series of tables, he presents to us the various accommodations provided for the sick in the prisons, and penitentiaries of Norway. At the same time it must be a subject of regret, that the committee have received from certain goals, documents so unsatisfactory, that perfect accuracy in this statistical account could not be obtained. The attention of the committee was particularly directed to the ascertaining the average number of sick in each prison, the number of visits paid to them weekly by the medical attendant, the proportion of deaths and recoveries, the effect that the situation, aliment, employment, &c., appeared to have upon the health of the inmates, and, finally, the number of cases of insanity or suicide which had occurred, and if these could be traced to have any connection with the peculiar discipline of the gaol. The number of beds in, and the size of each infirmary within the precincts of the prisons are also required to be given, that the amount of cubic feet of air allotted to each individual may be accurately determined. Nearly sixty pages of Dr. Holst's essay are occupied by these statistical accounts, which from their complex nature, are incapable of analysis, and from the imperfect returns upon which they are based, cannot claim the attention they would otherwise ensure. The author notices in the next place, the deficiencies which call for remedy in the Norwegian prisons, as far as regards the arrangements for the reception of sick convicts, and points out the results of such imperfect measures as have been hitherto adopted. In all, with the single exception of the rude prison of Vardoehuus, the sick man is immediately separated from his fellow prisoners. In general the height of the rooms used as infirmaries is much to be complained of, as in Vardoehuus they are only seven feet high, and in Bergen and Trondhjem only average ten. The Norwegians are not in general fond of bathing, and we therefore are not surprised to find that baths either warm, tepid, or cold, have only been provided in one or two of these localities.

Until a very recent period, the appointment of physician to the gaol has been held often by men not duly qualified to practise, and no Journals of the cases under their care are now forthcoming to satisfy the enquiries of the commission. Nor is the medical attendant obliged by the present rules, to visit daily the patients under his charge; in the prisons of Fredriksteen and Vardoehuus they only enter within the walls, when sent for by prisoners dangerously ill.

Another great irregularity, and a fruitful source of disease under the present system, is the little care bestowed upon the inspection of prisoners at their first arrival within the precincts of the gaol. It appears that this most necessary duty is often delayed from day to day, and that thus many contagious disorders may be, and are introduced among the inmates.

In order to ascertain as far as possible the results of these arrangements for the sick in the various prisons of Norway, the commission proposed in addition the following questions to the medical attendants:

What are the most frequent disorders among the convicts, and what their probable cause? What age, what sex is most liable to these? Can any of the trades, &c., exercised within the precincts of the gaol, be regarded as a cause of ill health? Has insanity showed itself in any of the prisoners in consequence of confinement? and, lastly, do the convicts leave the prisons in the enjoyment of better health than when they entered its gates?

The medical attendants of the gaols were also requested to transmit to the committee as complete an account as possible, of the arrangements under their charge for the reception and care of the sick prisoners, and to note carefully the diseases of which they died, or under which they suffered, as well as the average number of days of sickness in the year, in relation to the number of convicts confined within the walls of the prison.

The answers received to these inquiries of the commissioners tend to show that the diseases most frequent among the slaves are, catarrhal fevers, agues, erysipelas, frost bites, ulcers, itch, and in Vardoehuus, scurvy. The intermittent fevers occur only at Fredrikstad and Agershuus; in both these places the gaol is surrounded by ditches of stagnant water. Erysipelas appears chiefly upon the legs and ancles of the prisoners, and then, no doubt, is occasioned by the constant irritation of the iron rings which encircle them. The labour to which prisoners are subjected in Norway does not seem in any way prejudicial to health, unless, perhaps, the constant exposure, with insufficient clothing, in the open air, may give rise to numerous catarrhal and inflammatory attacks. During the prevalence of the cholera in Norway, the prisons did not escape the scourge; but, though the number of cases in the slave-houses far exceeded those in the towns, the mortality does not appear to have been greater than among the free population of the country.

It is a remarkable fact that the number of those who became affected with insanity during their abode in the gaols, was far less, on an average, than among the free inhabitants of the towns or country in Norway. Almost all the medical men concur in stating, as an undoubted fact, that the majority of the prisoners leave their place of confinement in better health than when they entered it.

Professor Holst next proceeds to illustrate, by admirably constructed tables, the information which has been obtained regarding the proportion of sick in each prison to the healthy inmates. The general average of the whole six *slave-houses*, appears to be 1 : 15, while each sick person was, on an average, detained twenty-five days in the infirmaries. In the penitentiaries the former were only as 1 : 29, and the average days of illness for each sick prisoner only 18.

The mortality in all the six slave-houses has been found to be as 1 : 51, and in the penitentiaries as 1 : 49; while the general mortality among the free population of Norway is 1 : 51. We thus are brought to the somewhat startling conclusion that the proportion of deaths in Norway is least among those whose crimes are visited with the severest chastisements:

"We should certainly," remarks Dr. Holst, "have expected the contrary, when we consider the unhealthy position of many of the *slave-houses*, the narrow space allotted to each prisoner, the age of those detained, and the filthy mode of life permitted in gaols, and we can only explain the greater mortality of the penitentiaries by the fact, that in the latter the prisoners work in close and ill-ventilated rooms, while the slaves are chiefly employed in hard labour in the open air."

Out of 10,867 prisoners, nine have committed suicide during the last ten or twelve years, and seven have perished by accident; six of these having been drowned by the upsetting of a boat in the neighbourhood of Trondhjem.

From a careful comparison of the present statistical tables, with those referred to in a former number of this Journal, we find that the mortality in the Norwegian prisons has been considerably less than in the gaols of other countries, under the older systems of prison discipline, and all will concur in the hope expressed by Dr. Holst, that when the deficient arrangements, now in force, have been improved and altered by the commission, we may expect a much greater reduction of deaths among the prisoners. It were indeed greatly to be desired, that all statistical accounts could be drawn up with the careful attention which has evidently been bestowed upon these investigations of Professor Holst.

